# *Ageratina adenophora* Inhibits Spleen Immune Function in Rats via the Loss of the FRC Network and Th1–Th2 Cell Ratio Elevation

**DOI:** 10.3390/toxins13050309

**Published:** 2021-04-26

**Authors:** Zhihua Ren, Pei Gao, Samuel Kumi Okyere, Yujing Cui, Juan Wen, Bo Jing, Junliang Deng, Yanchun Hu

**Affiliations:** Key Laboratory of Animal Diseases and Environmental Hazards of Sichuan Province, College of Veterinary Medicine, Sichuan Agricultural University, Chengdu 611130, China; zhihua_ren@126.com (Z.R.); gaopeijune@163.com (P.G.); samuel20okyere@gmail.com (S.K.O.); yjchoi@163.com (Y.C.); juanwen881010@163.com (J.W.); jingbooo@163.com (B.J.); dengjl213@126.com (J.D.)

**Keywords:** *Ageratina adenophora*, spleen, CD3^+^ T-lymphocyte, FRC network, Th1/Th2 ratio

## Abstract

The objective of this study was to determine the impact of *Ageratina adenophora (A. adenophora)* on splenic immune function in a rat model. Rats were fed with 10 g/100 g normal feed and an experimental feed, which was composed of 3:7 *A. adenophora* powder and normal feed for 60 days. On days 14, 28, and 60, subsets of rats (*n* = 8 rats/group/time point) were selected for blood and spleen tissue sample collection. The results showed that the proportion of CD3^+^ T cells in the spleen was decreased at day 60 (vs. control). Also, mRNA and protein expression of chemokines CCL21 and CCL19 and functional protein gp38 in spleen decreased significantly versus the control at day 60. In addition, ER-TR7 antigen protein expression was also decreased at day 60. Levels of T-helper (Th)1 cells significantly increased, whereas those of Th2 cells decreased significantly versus the control at day 60 in spleen. The finding revealed that *A. adenophora* could affect splenic immune function in rats by altering the fibroblast reticulocyte (FRC) network, as well as by causing an imbalance in Th1/Th2 cell ratios. This research provides new insights into potential mechanisms of spleen immunotoxicity due to exposures to *A. Adenophora.*

## 1. Introduction

*Ageratina adenophora (Spreng.)* R. M. King et H. Rob. (aka Crofton weed) is an invasive plant in China and throughout the world [1]. In China, *A. adenophora* was first reported in Yunnan (at the China–Myanmar and Sino–Vietnamese borders) in the 1950s, and then spread rapidly across southwestern China [2]. *A. adenophora* contains a variety of structurally diverse chemicals, including (mono-, sesqui-, di-, tri-) terpenoids, phenylpropanoids, flavonoids, coumarins, sterols, and alkaloids [3,4,5]. Zhang et al. [6] also identified some quinic acid derivatives in this plant. Other studies have reported that essential oils are present in this plant, and have also found significant levels of agents like α-phellandrene (3.85%), limonene (0.56%), β-caryophyllene (2.38%), carotol (1.17%), and 1-napthalenol (17.5%) in the invasive plant [7].

As an invading organism, *A. adenophora* has a bad ecological impact [8], including toxic reactions in livestock, such as horses, cattle, and goats, that ingest it during grazing [9,10,11,12]. Among the effects reported are abnormal liver hematology, jaundice, hepatocyte necrosis, bile duct hyperplasia, liver oxidative stress, and inflammation [13,14,15]. Other studies have also reported toxicity in the spleen, with characteristics like abnormal spleen cell proliferation, increased apoptosis, and tissue oxidative stress damage [16,17,18]. However, *A. adenophora* also have ethno-pharmacological uses, such as anti-malaria, anti-inflammation, analgesic, and wound healing [19]. Many traditional groups use this plant for treating various diseases, but the use of *A. adenophora* and its extracts poses a high risk of toxicity to humans due to incorrect dose administration and pre-clinical testing in the traditional medicine setup. In addition, humans also get exposed to the plant when some of their toxic metabolites get into food crops on the field. Therefore, improper dose administration could lead to some of the toxic findings mentioned above; hence, proper dose administration and trials are necessary to reduce the toxicity caused by this invasive plant.

Recent research has suggested that the main toxic components of *A. adenophora* are its sesquiterpenoids, such as 9-oxo-10,11-dehydro-agerophorone (ODA), 2-deoxo-2-(acetyloxy)-9-oxo- ageraphorone (DAOA), and 9-oxo-agerophorone (OA) [20,21,22,23]. Those authors not only detected each of these sesquiterpenes, but also found that they induced heap- and immunotoxicity. Meanwhile, Okyere et al. [24] reported that ODA (Euptox A) caused apoptosis and G_0_/G_1_ cell arrest of hepatocytes via an induced accumulation of reactive oxygen species (ROS). Mo et al. [17] reported that ODA induced G_1_ arrest and autophagy in mouse splenocytes through p38 MAPK- and PI3K/Akt/mTOR- mediated pathways. Nevertheless, other potentially toxic substances in *A. adenophora* have not been studied fully for their general toxicity, or their immunotoxic potentials in particular. Here we mainly focus on its toxicity to the spleen. There have been no detailed reports about the damage of *A. adenophora* to the spleen function recently.

The spleen is an important secondary lymphoid organ (SLO) in the body, and the T lymphocytes are important immune cells in the spleen. The activation, as well as the homing and recirculation between the blood and lymphatic organs of T lymphocytes, play a role in immune surveillance and clearance [25]. This process in the spleen is regulated by chemokines secreted by splenic stromal cells. Fibroblast reticulocyte (FRC), consisting of the reticular fiber network, is a kind of stromal cell in T cell zones of the white pulp. It can secrete high concentrations of chemokines CCL21 and CCL19 to guide T cell migration [26,27]. The transmembrane protein gp38 is a specific marker that can be used to locate T cell zones in the spleen and quantify the number of FRC cells [28,29,30]. ER-TR7 antigens secreted by FRC accumulate outside FRC cells and participate in the construction of FRC networks [31,32]. Th1 and Th2 are subsets of Helper T (Th) cells. Normally in healthy body system, Th1 and Th2 cells are in a dynamic equilibrium state, and they inhibit each other in activation and function to maintain the body’s cytokine microenvironment homeostasis [33].

Accordingly, in this study, the effects and potential mechanisms of action on the spleen and associated immune responses focusing on the FRC network and Th1–Th2 cell equilibrium were evaluated in rats fed *A. adenophora* leaf powder in their diets.

## 2. Results

### 2.1. Effect of A. adenophora Exposure on Rat Weight and Spleen

Rats were provided *A. adenophora*-supplemented diets for 60 days, and the effects on their body weight (per two weeks) (Table 1) and spleen weight were evaluated (days 14, 28, and 60) (Table 2). Over the course of the study, there were no significant changes in amount of feed consumed (vs. control values) by rats in the *A. adenophora* groups (data not shown). The results showed that the body weight of the *A. adenophora* rats decreased significantly at day 28 (*p* < 0.05), and days 42 and 60 (*p* < 0.01) versus the control. Spleen weights significantly increased versus the control values at days 28 (*p* < 0.05) and 60 (*p* < 0.01). The spleen–body ratios significantly increased versus the control values only at day 60 (*p* < 0.01).

**Figure 1:**
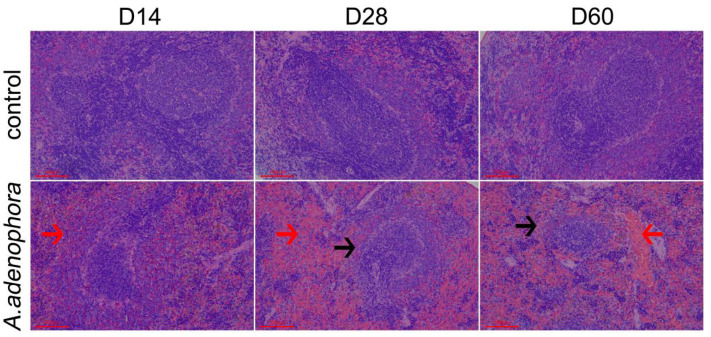
Representative photomicrographs of H&E-stained spleens. Histological structure of control rat tissues was normal. Spleens of treated rats displayed different degrees of pathological damage. Day 14: slight congestion of red pulp (red arrow). Day 28: severe congestion of red pulp (red arrow), white pulp atrophy (black arrow). Day 60: white pulp atrophy (black arrow), partial bleeding in the red pulp (red arrow). Scale bars = 100 μm.

Histologic analyses indicated that the control rats developed no pathological changes; in contrast, the *A. adenophora*-treated hosts did show some pathological changes at all the timepoints (Figure 1). Microscopic examination of the tissues showed that at day 14, the spleens of treated rats were slightly congested (i.e., blockage of the blood flow through the splenic vasculature and pooling of blood in the red pulp, which leads to dilated veins and sinuses); at day 28, spleens were congested, and there was white pulp atrophy (Figure 1). At day 60, white pulp atrophy was also found to be significant (vs. control). In the red pulp, hematopoietic cells were increased, the splenic sinus was dilated and congested, and there were indications of local bleeding in several areas (Figure 1).

### 2.2. A. adenophora Exposure Induced Abnormal CD3^+^ T-Lymphocyte Levels in the Blood and Spleen

**Figure 2:**
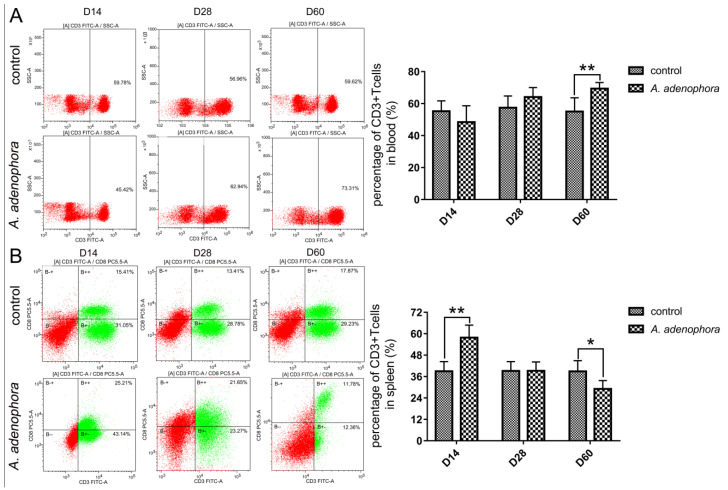
*A. adenophora* treatment increased the proportion of CD3^+^ T cells in the blood and decreased their proportion in spleen. Proportion of CD3^+^ T cells in the (**A**) blood and (**B**) spleen. Values shown are means ± SD, *n* = 8/group/timepoint. Value significantly different from time-matched control. Bars showing * and ** are significantly different at * *p* < 0.05 or ** *p* < 0.01.

In order to observe the effects of *A. adenophora* on CD3^+^ T-lymphocytes, the levels of these CD3^+^ cells in the spleen and blood of rats in each group were assessed by flow cytometry. As shown in Figure 2, although the proportion of CD3^+^ T cells in the control rats changed in both compartments over time, the net change across the three timepoints was not significant. In contrast, these proportions changed significantly in the *A. adenophora*-exposed rats. Specifically, the blood levels of CD3^+^ T cells increased significantly versus the control at day 60 (*p* < 0.01; Figure 2A), whereas the proportion of these cells in the spleen increased significantly at day 14 (*p* < 0.01), and then decreased at day 60 (*p* < 0.05) compared to the control rat values (Figure 2B).

### 2.3. A. adenophora Exposure Caused Disruption of Splenic Fibroblast Reticulocyte (FRC) Network

**Figure 3:**
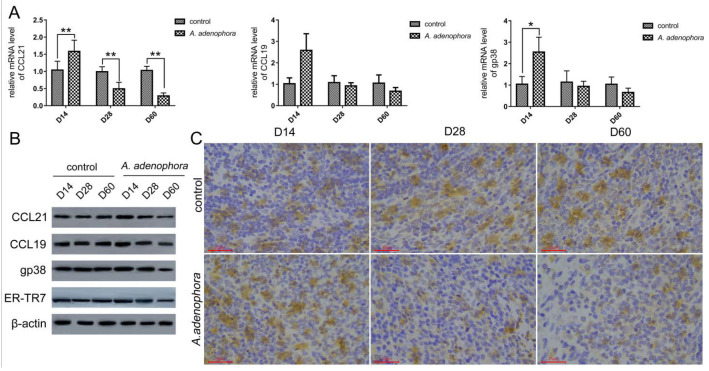
*A. adenophora*-mediated loss of the splenic FRC network. (**A**) Relative mRNA expression of genes for *CCL21*, *CCL19* and *gp38* in spleen of rats in each group. Data shown are means ± SD, *n* = 8/group/timepoint, Bars showing * and ** are significantly different, at * *p* < 0.05 or ** *p* < 0.01. (**B**) Western blot results for splenic levels of CCL21, CCL19, gp38, and ER-TR7 in each group. (**C**) Immunohistchemical analyses of gp38 levels in spleen of rats in each group.

To explore the potential underlying reasons for the noted reductions in splenic CD3^+^ T cells, levels of CCL21 and CCL19 chemokines, as well as gp38 and ER-TR7 antigens, were evaluated in samples of splenic tissues via qRT-PCR, Western blotting, and immunohistochemistry. The results showed that the mRNA levels of *CCL21* in *A. adenophora*-treated rats increased significantly at day 14 (*p* < 0.01), but decreased at days 28 and 60 when compared to control host values (*p* < 0.01) (Figure 3A). In a similar manner, the mRNA levels of gp38 in the *A. adenophora* rats increased significantly (vs. control value) at day 14 (*p* < 0.05), but decreased versus the control (not significant; *p* > 0.05) by day 60. Lastly, splenic mRNA levels of *CCL19* in the *A. adenophora* rat group increased compared to the control at Day 14, but this change was not significant (*p* > 0.05).

Western blotting (WB) and immunohistochemical analyses showed that the splenic protein levels of CCL21 in *A. adenophara*-treated rats increased and decreased (not significantly; *p* > 0.05) at days 14 and 28 (vs. control host values), respectively (Figure 3B,C and Table 3). However, the protein level of CCL21 decreased significantly at day 60 (vs control host value) (*p* < 0.01). CCL19 and gp38 protein levels in *A. adenophara*-treated rats increased significantly at Day 14 (*p* < 0.05 and *p* < 0.01, respectively), then finally decreased significantly at day 60 (vs. control host values) (*p* < 0.01 and *p* < 0.05, respectively). Furthermore, the protein levels of ER-TR7 antigen in the *A. adenophora*-treated rats increased and decreased (not significantly; *p* > 0.05) at days 14 and 28 (vs. control host values) respectively; however, protein levels decreased significantly at Day 60 (vs. control host value) (*p* < 0.05). Finally, the immunochemistry results showed that the protein levels of the gp38 in the treated rats increased significantly at Day 14 and later reduced at day 60 (*p* < 0.05), which was similar to the results of the WB.

### 2.4. A. adenophora-Induced Splenic Th1 and Th2 Cell Imbalance and Increased Th1-Type Response

**Figure 4:**
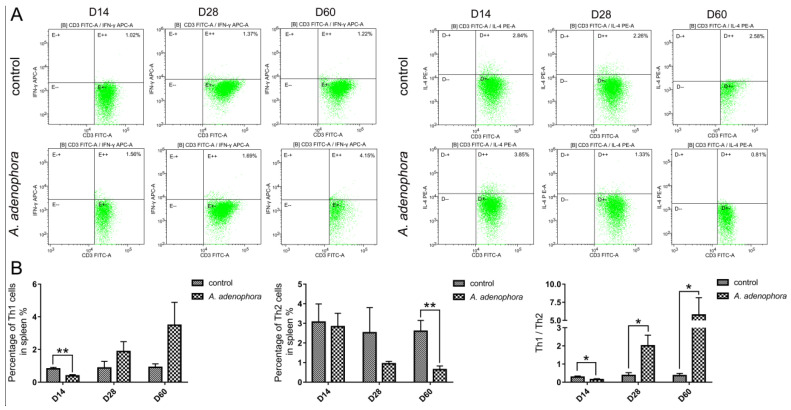
Flow cytometry results for Th1 and Th2 lymphocyte levels in spleens. (**A**) Representative photographs of flow cytometry of spleens (**B**) Percentage of Th1 and Th2, as well as the Th1/Th2 ratio. Data shown are means ± SD, *n* = 8/group/timepoint. Bars showing * and ** are significantly different: * *p* < 0.05 or ** *p* < 0.01.

To assess the effects of *A. adenophora* on Th1 and Th2 cells (based on CD4^+^) and related factors in the spleen, flow cytometry was employed. As shown in Figure 4A, *A. adenophora*-treated rats had significantly different levels of Th1 and Th2 cells compared to the spleens of time-matched controls. The analyses revealed that, compared to the spleens of control rats, Th1 cell levels increased at day 60 (*p* > 0.05), whereas Th2 cells decreased significantly (*p* < 0.01) (Figure 4B), resulting in a shift in the splenic Th1/Th2 ratio.

Analyses of mRNA expression of the *T-bet* and *IFN-γ* (important transcription factor and cytokine of Th1 cells, respectively) genes did not indicate any significant changes within the first 28 days of treatment (Table 4); however, the levels of both were significantly increased versus the control by Day 60 (*p* < 0.01 and *p* < 0.05, respectively). Expression of the Th2 cell transcription factor *GATA3* and representative *IL-4* also did not change significantly within the first 28 days; however, their mRNA levels decreased significantly versus the control by day 60 (*p* < 0.05) (Table 4). Evaluation of the protein forms (ELISA) of IFN-γ and IL-12 in spleen homogenates revealed that levels of both were increased significantly versus control spleen values at day 60 (*p* < 0.01) (Table 5). In contrast, protein forms of Th2-representative IL-4 and IL-10 decreased significantly (vs. control) at day 60 (*p* < 0.01).

## 3. Discussion

Researchers have studied the main toxic substances, target organs, and toxic effects of *A. adenophora*, and found that *A. adenophora* mainly affects the liver and spleen [21]. This result was in agreement with other studies of *A. adenophora* leaf toxicity [11,15,18,34]. In the current study, the leaves of *A. adenophora* were evaluated for their ability to induce toxicity in the spleen of hosts ingesting them (as a powder) in their standard diet. Untoward effects on spleen weight and area, as well as the cellularity of splenic white and red pulp were noted, confirming earlier findings that *A. adenophora* could cause splenic damage when ingested [16]. Again, many pathways of toxicity of *A. adenophora* on the spleen have been reported recently; however, this study provides a novel mechanism of action that *A. adenophora* uses to cause spleen dysfunction, in order to be a guide in drug development for reducing the effects of this plant on the immune organ (spleen) when ingested into the body.

The expression levels of gp38 and ER-TR7 antigen can be used to reflect the state of the FRC network in splenic T cell zones. Furthermore, the constitutive chemokines CCL21 and CCL19 secreted by FRC affect the number of T cells entering the spleen. The present study found that changes in the expression of both gp38 and ER-TR7 were consistent with those in levels of CCL21 and CCL19 in the *A. adenephora*-treated rats. From these results, we suggest that the loss of the normal FRC network in the spleen causes a decrease in the formation/release of important chemokines, just as was reported by Bajénoff et al. [26]. Therefore, the increase in the total period of ingestion of the powdered leaf led to an increasing impact on the splenic FRC network, leading to a reduction in CCL21 and CCL19, and ultimately shifting the T cell levels due to impaired homing of T cells into the spleen.

Normally, the status of Th1 and Th2 cells in a body is in a state of equilibrium; this is important for immune environment homeostasis, and an imbalance among these cells may cause abnormal immune responses [35,36,37]. Of the three timepoints in this experiment, the number of Th1 and Th2 cells in the spleen was changed only at day 60 in the *A. adenephora*-treated rats, with the proportion trending toward Th1 cells. In addition, the expression levels of mRNA for the master transcription factor and cytokine (T-bet and IFN-γ), reflecting Th1 type responses, increased significantly at Day 60, while GATA3 and IL-4 (representing Th2 type responses) concurrently and significantly decreased at the same time point. However, in the treated hosts, the protein levels of IFN γ and IL-12 in the spleens increased significantly (vs. control) at day 60, while those of IL-4 and IL-10 decreased significantly. Thus, it would seem that the Th1/Th2 cell balance in the spleen could be altered (even potentially long-term) as a result of continual ingestion of *A. adenophora*.

The mechanism behind these observations is not yet clear. It seems that when *A. adenophora* is ingested into the rat body, it induces a persistent inflammatory response in the spleen. This in turn could lead to activation and promotion of T cell immunity [38,39,40], which then causes further imbalance and eventually splenic dysfunction. It has also been reported that *A. adenophora* caused oxidative stress and inflammatory pyrolysis in the spleen [18]. In this experiment, it was found that, the loss of the FRC network and changes in levels of local immune response factors in the spleen were part of the overall inflammatory damage caused by *A. adenophora.* Also, even though the levels of CCL21/CCL19 and functional proteins showed similar trends as CD3^+^ splenic T cells, and supported the observed reduction on day 60, CD3^+^ blood T cells increased at day 60 (vs. control host value). We speculated that this inconsistency may be because *A. adenophora* might lead to abnormal T cell distribution in the exposed host. However, this needs further studies to ascertain this occurrence. In addition, the impact of *A. adenophora* on the immune system also needs to be further explored. Therefore, the destruction of the cells in the spleen could lead to improper function of the spleen, leading to the emergence of infectious and other immune-related diseases in humans and animals. Also, spleen dysfunction decreases the production of memory T cells, thereby making it difficult to heal infections that are reappearing [41].

Although our group did not perform chemical analyses of the *A. adenophora* samples, the analyses performed in other studies have previously revealed an abundance of three important sesquiterpenes, i.e., 2-deoxo-2-(acetyloxy)-9-oxoageraphorone (DAOA), 9-oxo-agerophorone (OA), and 9-oxo-10,11-dehydro-agerophorone (ODA, Euptox A), in the plant leaves. Levels of each in the leaves are far higher than in any other part of the plant, with 0.63–1.99% of the mass percentage found in dried leaves [16,42,43]. Based on the toxicology literature, it would seem plausible these sesquiterpenes could be possible causative agents for the splenic immunomodulatory effects noted here. A study by Mo et al. [17] stated that Euptox A induced G_1_ arrest and autophagy among the splenocytes of mice who had ingested the toxin. Ouyang et al. [16] specifically reported that both DAOA and OA caused alterations in the histopathology of the spleens of mice that had been exposed to these toxic compounds.

Clearly, the immunotoxicity of specific agents present in *A. adenophora* (including the above-noted sesquiterpenes) and their effects on the spleen should be investigated individually. Such future studies would help in determining the main toxic substance(s) that cause immune/splenic dysfunction after repeated ingestion of this plant.

## 4. Conclusions

**Figure 5:**
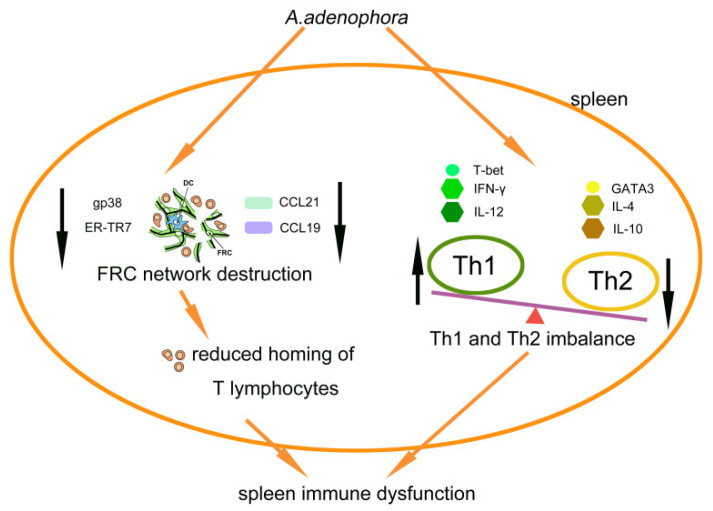
*A. adenophora* exposure induces “chronic” inflammation in the spleen, destruction of the FRC network, and dysregulation of Th1/Th2 ratio. Together, these changes likely lead to insufficient T cell migration and microenvironmental changes in the levels of Th1- and Th2-associated factors in the spleen.

The consumption of the leaves of the *A. adenophora* plant by rats induce significant damage in the spleen, including a loss of the FRC network, shifts in the Th1/Th2 cell ratio, and alterations in the expression of Th1 and Th2 cell-related factors in the spleen (Figure 5). These changes might suggest some underlying mechanisms for any immunotoxicity associated with ingestion of *A. adenophora*. Furthermore, this study will also provide clues about targets to treat, in order to potentially mitigate certain toxic aspects associated with inadvertent consumption of this invasive plant.

## 5. Material and Methods

### 5.1. Plant Material

For the studies, samples of *A. adenophora* were collected from Dechang in Sichuan Province (102°15′20″ E and 27°20′11″ N; elevation = 2152 m). The samples were confirmed as *A. adenophora* by Prof. Chao Hu, Department of Botany, Sichuan Agricultural University. The samples were air-dried and then ground into a fine powder using a CW700 grinding machine (Pharmaceutical Machinery Co., Shan Dong, China). Sizing analyses showed that the mean diameter of the powder particles was 500 μm. For the experimental feed, *A. adenophora* powder was mixed uniformly with normal feed powder (Chengdu Dossy Experiment Animal Co., Ltd, Chengdu, China; Table 6) in a ratio of 3:7. Both normal and experimental feed were shaped into a pellet with a cylindrically-shaped tube, with a diameter of 2 cm and height of 5 cm. as in Sun et al.’s study [18].

### 5.2. Animals and Experimental Design

Male Sprague–Dawley (SD) rats (7 weeks of age, ≈200 g) were obtained from the Dossy Experiment Animal Co., Ltd (Chengdu, China). All rats were housed in specific pathogen-free facilities, maintained at 22–24 °C with 40–60% relative humidity and a 12 h light/dark cycle. After 7 days of adaptation, the rats were randomly allocated into two groups, i.e., a control (*n* = 24) that was fed normal feed at 10 g/100 g BW for 60 days, and an *A. adenophora* group (*n* = 24) that received 10 g/100 g BW of the experimental feed for the same period. This dose was based on a pilot experiment that indicated that 30% *A. adenophora* leaf powder added to normal feed for the same total period of time did not affect rat feed intake [15]. Water was given ad libitum to all treatments.

The rats were observed over the course of the entire 60-day period; body weight, behavior, illness, and mortality parameters were taken/recorded daily. Subsets of the rats were euthanized by CO_2_ asphyxiation on days 14, 28, and 60 (*n* = 8 rats/group/timepoint); all rats were fasted for 12 h prior to being euthanized. At necropsy, blood from the abdominal aorta was collected into anticoagulant-coated tubes; the spleen was then aseptically removed and processed as indicated in the assays outlined below. This study was approved by Sichuan Agricultural University Animal Care and Use Committee (approval no. 2012-024). All animal operations and procedures were conducted according to the approved guidelines, and were in accordance with the international Guide for the Care and Use of Laboratory Animals.

### 5.3. Histological Analysis

After weighing the isolated spleen, samples (1/2 of each spleen collected at necropsy) were fixed in 10% buffered neutral formalin solution, and then dehydrated in graded (70–100%) alcohol. After being cleared in xylene, each sample was embedded in paraffin, and 5 µm-thick sections were prepared. For analyses, some samples were stained with hematoxylin and eosin (H&E) dye, and then evaluated using a light microscope. A total of eight rats/group were evaluated; for each rat, three stained slices were examined. Other sections were used for immunohistochemical staining.

### 5.4. Flow Cytometry Analysis

To evaluate CD3^+^ T cells in the blood, aliquots of the whole blood (100 μL/rat) were treated with the FITC-conjugated anti-CD3 (#559975 for CD3^+^ T cells) at the manufacturer-recommended level, and then incubated in the dark for 30 min at 4 °C. To each tube, 2 mL Lysing Solution (ab186198, Abcam, London, United Kingdom) was then added to eliminate any red blood cells present. The samples were incubated for 10 min at 25 °C, and then the contents were centrifuged at 250× *g* for 5 min. The resulting supernatant was discarded, and then 400 μL PBS was added to the RBC-free samples. After re-suspension, the cells were immediately analyzed using a CytoFLEX flow cytometer (Beckman Coulter, Shanghai, China).

To evaluate CD3^+^ T cells, as well as T_H_1 and T_H_2 in the spleen, samples of splenic tissue (150 mg/rat) were cut into pieces and then passed through a 300-mesh nylon screen using a syringe plunger. After repeated washing of the mesh with phosphate-buffered saline (PBS, pH 7.4), the cell isolate was centrifuged at 250× *g* for 5 min. After re-suspension in PBS, cells were counted using a hemocytometer, and the density adjusted to 10^6^ live cells/mL. From this, a 100-µL aliquot of the suspension was transferred into a sterile tube, for which a specific anti-rat marker antibody was added (at the manufacturer-recommended level). The sample was then left to sit in the dark at 4 °C for 30 min. Antibodies used included fluorescein isothiocyanate (FITC)-conjugated anti-CD3 (#559975 (for CD3^+^ T cells), Becton Dickinson, San Jose, CA, USA), phycoerythrin (PE) anti-CD8 (#558824, Becton Dickinson), AlexaFluor (AF)-647-anti-IFN-γ (#507810, Biolegend, San Diego, CA, USA; for T_H_1), or PE anti-IL-4 (#511906, Biolegend; for T_H_2). After incubation, cells were immediately analyzed in a CytoFLEX flow cytometer (Beckman Coulter). All data were visualized and analyzed using Kaluza.2.1 software (Becton Dickinson Beckman Coulter, Brea, CA, USA). A minimum of 10,000 events/sample was acquired.

### 5.5. Extraction of Total RNA and Quantitative Real Time RT-PCR (qRT-PCR)

Samples of splenic tissue (30 mg/rat) were snap-frozen with liquid N_2_, and then immediately ground into powder using a ceramic mortar. The total RNA from each sample was extracted with an Animal Total RNA Isolation Kit (Sagon Biotech, Shanghai, China) according to manufacturer instructions. After confirming the isolated RNA concentration and purity using a NanoDrop One system (Thermo Fisher Scientific, Waltham, MA, USA; OD260/280 ≈ 1.9–2.0), triplicate aliquots (each 1 µg) were removed, loaded into wells, and the cDNA of each prepared using a PrimeScrip RT reagent kit (Takara, Tokyo, Japan). Thereafter, qRT-PCR was performed using a SYBR Premix ExTaq (Takara, Tokyo, Japan) and a CFX96 thermal cycler (BioRad, Hercules, CA, USA). The primers used to analyze the genes of interest were designed from NCBI’s genBank and are shown in Table 7. The relative gene expression in each sample was normalized to an internal control (*β-actin*); data analysis was done using the 2^−ΔΔCt^ method [44]. All samples were evaluated in triplicate.

### 5.6. Enzyme-Linked Immunosorbent Assay (ELISA)

Samples of splenic tissue (0.2 mg/rat) were homogenized on ice in PBS (ratio of 1:9, *w/v*) using a glass pestle. The suspension was then centrifuged at 2000× *g* for 10 min at 4 °C. The resulting supernatants were each evaluated for levels of interleukin (IL)-12p70, IL-4, IL-10, and interferon (IFN)-γ, using commercial ELISA kits (Jingmei Biological Technology, Jiangsu, China), following manufacturer protocols. All levels in samples were determined by extrapolation from standard curves generated in parallel in each kit. All data are reported in terms of pg/mL. The level of sensitivity of each kit was 0.1 pg/mL for each cytokine.

### 5.7. Western Blot Analysis

Samples of splenic tissue (50 mg/rat) were extracted using a Tissue/Cell Total Protein Extraction Kit (Sagon Biotech, Shanghai, China). The total protein concentration in each lysate was measured (using a BCA method [45]), and then aliquots containing 40 µg protein were loaded into wells of dedicated 10–15% polyacrylamide gels (SDS-PAGE; one for each protein of interest) for separation. Post-resolution, each gel’s contents underwent Western blotting using standard incubation, washing, and detection protocols. In brief, after the resolved proteins of each gel were electrotransferred to a polyvinylidene difluoride membrane, each membrane was blocked for 1 h at 25 °C in a 5% non-fat milk/TBST (Tris-buffered saline containing 0.1% Tween-20) solution. After gentle rising with TBST, each membrane was incubated for 12 h at 4 °C in a solution of TBST containing one specific rabbit or mouse anti-rat monoclonal antibody, at a level recommended by the respective manufacturer. The antibodies used were anti-rat CCL19 (Absin, abs124189, Shanghai, China), anti-CCL21 (abs136201), anti-podoplanin/gp38 (sc- 166906, Santa Cruz Technology. Santa Cruz, CA, USA), or anti-fibroblast/lymphoid extracellular matrix marker (ER-TR7) (sc-73355, Santa Cruz).

After the incubation, each membrane was gently rinsed with TBST, and then incubated for 1 h at 25 °C in TBST containing horseradish peroxidase (HRP)-conjugated anti-rabbit (or mouse as required) secondary antibody (Santa Cruz), in order to detect the presence of the primary antibody. Equal loading of proteins was confirmed by measures of β-actin expression (using corresponding primary antibody). After a final TBST rinse, immunoreactivity was visualized by membrane incubation in BeyoECL Moon solution (Beyotime, Shanghai), and then analysis of chemiluminescence in a ChemiDOC MPimaging system (Bio-Rad, Hercules, CA, USA). All results for band intensity were analyzed using ImageJ 1.8.0 (NIH, Bethesda, MD, USA).

### 5.8. Immunohistochemical Staining

Tissue sections were coated with 3% hydrogen peroxide solution for 10 min at 25 °C to inactivate endogenous peroxidases. Thereafter, the slides were placed in 10 mM citrate buffer and heated for 20 min at 95 °C for antigen retrieval. After this, each slide was placed in 5% goat serum/PBS (blocking; Sigma, St. Louis, MO, USA) for 30 min, and then incubated in a solution of biotin-conjugated, anti-gp38 antibody (in PBS; Sagon Biotech, Shanghai, China) overnight at 4 °C. After extensive washes with PBS, each section was then placed in a solution of PBS-biotin-conjugated, anti-rabbit antibody for 15 min at 25 °C. After extensive washes with PBS, the bound antibody was visualized using diaminobenzidine (DAB). All slides were examined using a CX22 microscope (Olympus, Tokyo, Japan), and relative antibody staining was quantified visually.

### 5.9. Statistical Analysis

All results are shown as means ± SD. For data that displayed a normal distribution after a Shapiro–Wilk Test, a one-way analysis of variance (ANOVA) was used to detect differences among the groups. Data sets that did not display normal distribution were analyzed by non-parametric tests (Kruskal–Wallis test). In the experiment, the *A. adenephora* was compared to the control group at each time point. All data were analyzed using SPSS 22 (SPSS Inc., Chicago, IL, USA). In all cases, a *p*-value < 0.05 or *p*-value < 0.01 was considered significant.

## Figures and Tables

**Table 1:** Changes of body weight in rat/g.

Group	D0	D14	D28	D42	D60
Control	225.99 ± 7.57	276.03 ± 9.33	312.77 ± 11.54	347.71 ± 15.91	366.60 ± 15.65
*A. adenophora*	227.37 ± 11.70	239.14 ± 16.97	207.27 ± 10.29 *	199.20 ± 14.64 **	190.78 ± 11.86 **

Data is presented as mean ± standard deviation (SD). All values in column with * are statistically different at *p <* 0.05, and ** indicates statistically different at *p <* 0.01.

**Table 2:** The absolute and relative weight of the spleen.

Time	Spleen Weight/g		Spleen–Body Ratio %	
	Control	*A. adenophora*	Control	*A. adenophora*
D14	0.53 ± 0.092	0.53 ± 0.055	0.19 ± 0.035	0.23 ± 0.040
D28	0.62 ± 0.092	0.76 ± 0.058 *	0.20 ± 0.033	0.37 ± 0.041
D60	0.66 ± 0.085	1.14 ± 0.160 **	0.18 ± 0.019	0.60 ± 0.110 **

Data is presented as mean ± standard deviation (SD). All values in row with * are statistically different at *p <* 0.05, and ** indicates statistically different at *p <* 0.01.

**Table 3:** Protein levels of CCL21, CCL19, ER-TR7, and gp38 in the spleen.

Factors	D14		D28		D60	
	Control	*A. adenophora*	Control	*A. adenophora*	Control	*A. adenophora*
CCL21	0.72 ± 0.20	1.05 ± 0.29	0.67 ± 0.28	0.43 ± 0.18	0.78 ± 0.03	0.21 ± 0.07 **
CCL19	0.99 ± 0.24	1.29 ± 0.08 *	0.94 ± 0.08	0.79 ± 0.16	0.90 ± 0.08	0.46 ± 0.07 **
ER-TR7	1.10 ± 0.15	1.12 ± 0.26	1.00 ± 0.32	0.77 ± 0.29	0.94 ± 0.13	0.38 ± 0.08 *
gp38 (WB)	0.61 ± 0.20	1.21 ± 0.32 **	0.63 ± 0.23	0.65 ± 0.25	0.65 ± 0.17	0.25 ± 0.05 *
gp38 (immune-chemistry)	0.22 ± 0.04	0.34 ± 0.04 **	0.22 ± 0.02	0.13 ± 0.01 **	0.21 ± 0.03	0.08 ± 0.01 **

Data was presented as mean ± standard deviation (SD). All values in row with * were statistically different at *p <* 0.05, and ** indicates statistically different at *p <* 0.01.

**Table 4:** Relative mRNA levels of T-bet, IFN-γ, GATA3, and IL-4 in the spleen.

Factors	D14		D28		D60	
	Control	*A. adenophora*	Control	*A. adenophora*	Control	*A. adenophora*
T-bet	0.96 ± 0.25	1.44 ± 0.42	1.05 ± 0.26	1.51 ± 0.31	1.09 ± 0.33	1.84 ± 0.74 **
IFN-γ	1.10 ± 0.43	1.37 ± 0.48	1.13 ± 0.49	2.67 ± 1.79	0.94 ± 0.22	3.95 ± 0.80 *
GATA3	0.92 ± 0.24	0.78 ± 0.09	0.99 ± 0.32	0.88 ± 0.29	1.03 ± 0.15	0.50 ± 0.09 *
IL-4	1.05 ± 0.34	1.17 ± 0.63	1.06 ± 0.45	0.76 ± 0.30	1.08 ± 0.32	0.54 ± 0.22 *

Data are presented as mean ± standard deviation (SD). All values in a row with * are statistically different at *p <* 0.05, and ** indicates statistically different at *p <* 0.01.

**Table 5:** Protein levels (ELISA) of IFN-γ, IL-12, IL-4, and IL-10 in the spleen (pg/mL).

Factors	D14		D28		D60	
	Control	*A. adenophora*	Control	*A. adenophora*	Control	*A. adenophora*
IFN-γ	1614.62 ± 32.34	1621.06 ± 87.80	1615.07 ± 112.19	1614.96 ± 38.27	1609.92 ± 97.87	1767.11 ± 66.85 **
IL-12	13.30 ± 0.97	13.84 ± 0.71	13.14 ± 0.84	14.08 ± 0.29	13.24 ± 0.82	15.27 ± 0.50 **
IL-4	89.20 ± 7.79	97.87 ± 4.71 *	85.07 ± 4.79	80.78 ± 5.84	89.32 ± 3.64	72.25 ± 3.82 **
IL-10	41.84 ± 3.11	37.40 ± 1.66*	41.42 ± 3.77	37.44 ± 2.20*	41.54 ± 2.95	34.69 ± 1.50 **

Data are presented as mean ± standard deviation (SD). All values in a row with * are statistically different at *p <* 0.05, and ** indicates statistically different at *p <* 0.01.

**Table 6:** Composition of the normal feed.

Ingredients	Content %
Water	10.00
Crude protein	18.00
Crude fat	4.00
Crude fiber	5.00
Crude ash	8.00
Calcium	1.50
Phosphorus	1.00
Lysine	0.82
Methionine + cystine	0.53

**Table 7:** Primers used for real-time PCR analysis.

Gene	Primer Sequence (5′→3′)	Sequence Number
IFN γ	F: GGCAAAAGGACGGTAACACG R: TCTGTGGGTTGTTCACCTCG	NM_138880.3
IL-4	F: AAGGAACACCACGGAGAACG R: CAGACCGCTGACACCTCTAC	NM_201270.1
T-bet	F: GTATGCCAGGGAACCGCTTA R: ATTGTTGGAAGCCCCCTTGT	NM_001107043.1
GATA3	F: CTCTTCCCTCCCAGCAGCCTAC R: AGTACCATCTCGCCGCCACAG	NM_133293.1
CCL21	F: ACAGGAAGCAAGAACCGAGC R: TCTGTCTGTTCAGTCCCCTTG	NM_001008513.1
CCL19	F: GTGCTAACGATGCGGAAGAC R: AGAGCTGGTAGCCCCTTAGT	NM_001108661.1
gp38	F: TAAGAGAGCTTCCACCTTGCC R: GGCTTGTCCCGTATCTTTCCT	NM_019358.2
β-actin	F: ACGGTCAGGTCATCACTATCG R: GGCATAGAGGTCTTTACGGATG	NM_031144.3

F: forward; R: reverse.

## Data Availability

The data presented in this study are available on request from the corresponding author.
